# Influence of tumor microenvironment on prognosis in colorectal cancer: Tissue architecture-dependent signature of endosialin (TEM-1) and associated proteins

**DOI:** 10.18632/oncotarget.2108

**Published:** 2014-06-16

**Authors:** Daniel J. O'Shannessy, Elizabeth B. Somers, Lakshmi K. Chandrasekaran, Nicholas C. Nicolaides, Jennifer Bordeaux, Mark D. Gustavson

**Affiliations:** ^1^ Department of Translational Medicine and Diagnostics, Morphotek Inc., Exton, PA; ^2^ HistoRx Inc. (A subsidiary of Genoptix Medical Laboratory, Inc.), Carlsbad, CA

**Keywords:** Endosialin, TEM-1, CRC, tumor microenvironment, prognosis

## Abstract

Tumor survival is influenced by interactions between tumor cells and the stromal microenvironment. One example is Endosialin (Tumor Endothelial Marker-1 (TEM-1) or CD248), which is expressed primarily by cells of mesenchymal origin and some tumor cells. The expression, as a function of architectural masking, of TEM-1 and its pathway-associated proteins was quantified and examined for association with five-year disease-specific survival on a colorectal cancer (CRC) cohort divided into training (n=330) and validation (n=164) sets. Although stromal expression of TEM-1 had prognostic value, a more significant prognostic signature was obtained through linear combination of five compartment-specific expression scores (TEM-1 Stroma, TEM-1 Tumor Vessel, HIF2α Stromal Vessel, Collagen IV Tumor, and Fibronectin Stroma). This resulted in a single continuous risk score (TAPPS: TEM-1 Associated Pathway Prognostic Signature) which was significantly associated with decreased survival on both the training set [HR=1.76 (95%CI: 1.44-2.15); p<0.001] and validation set [HR=1.38 (95%CI: 1.02-1.88); p=0.04]. Importantly, since prognosis is a critical clinical question in Stage II patients, the TAPPS score also significantly predicted survival in the Stage II patient (n=126) cohort [HR=1.75 (95%CI: 1.22-2.52); p=0.002] suggesting the potential of using the TAPPS score to assess overall risk in CRC patients, and specifically in Stage II patients.

## INTRODUCTION

Maintenance of epithelial tissues, including those involved in malignant diseases, requires interactions with neighboring cells, especially stromal cells. It has been well documented that the formation of solid tumors requires the proliferation of stromal cells to support cancer cell growth, invasion, and metastasis [1]. The stromal cell compartment comprises a heterogeneous mix of cells that are responsible for the formation of blood vessels as well as supporting a microenvironment comprised of fibroblasts and leukocytes. Stromal changes at the leading edge of invasive tissues include the appearance of myofibroblasts, which are cells that share several characteristics with fibroblasts and smooth muscle cells [2]. The coordinated growth and cross-talk between stromal cell components are critical for establishment of a microenvironment that can support the growth and maintenance of tumor cells. This cross-talk is mediated through direct heterotypic cell–cell contacts as well as through secreted molecules, comprising growth factors, cytokines, chemokines, extracellular matrix (ECM) proteins, proteinases, proteinase inhibitors, and lipid moieties [3,4]. Experimental animal models have demonstrated that cancer invasion is stimulated by stromal microenvironments similar to those present in wound healing [5]. This observation suggests that growth factors implicated in wound healing such as transforming growth factor-β (TGF-β) and platelet-derived growth factor (PDGF) may also play a role in altering the stromal host compartment in support of cancer [6]. In both wound healing and tumorigenesis, the fibroblast-to-myofibroblast transition marks the stromal alteration that leads to the biological functions of the lesion.

The stromal microenvironment is also important for supplying blood and nutrients to tumor cells via growth of new blood vessels, or angiogenesis, which is similarly critical for tissue growth, wound healing, and embryo development [6, 7]. As part of the angiogenic process, fibroblasts have been found to serve a vital role in secreting ECM proteins that are required for modeling and stabilizing the budding edge and vascular network of new blood vessels [7]. These proteins constitute a structural scaffold for proliferating endothelial and tumor tissues and, more importantly, provide support for the attachment of tumor cells. Tumor vasculature is also comprised of pericytes whose function is to stabilize endothelial cell-cell assembly that in turn provides support for the vessel lumen and blood flow to the tumor microenvironment [8]. In light of the critical relationship of tumor cells and stroma, anti-cancer strategies aimed at disrupting the stromal cell compartment, including suppression of angiogenesis, have been vigorously pursued [9].

Endosialin, also called Tumor Endothelial Marker-1 (TEM-1) or CD248, is one of several proteins that are localized to the tumor stromal compartment [10-12]. The protein was first discovered using a whole cell immune approach, whereby human fetal fibroblasts that have many characteristics similar to stromal cell fibroblasts, were used to immunize immunocompetent mice [13]. These efforts led to the development of an antibody called FB5 that recognized an antigen associated with tumor stroma. Years later, an independent effort identified cell surface markers on primary tumor endothelium via Serial Analysis of Gene Expression (SAGE). This research identified the TEM-1 gene product as the FB5 antigen [14]. Further examination of gene expression patterns in normal and neoplastic tissue have indicated up-regulation of TEM-1 expression in tumor neovessels within human colorectal cancer [10], breast cancer [15, 16], histiocytomas [17] as well as expression directly on tumor cells of mesenchymal origin including sarcoma [18,19] and melanoma [20]. Human TEM-1 expression has also been reported in highly invasive glioblastoma, anaplastic astrocytomas, and metastatic carcinomas [21]. Refined localization studies have found endosialin to be expressed on tumor associated pericytes and at the leading edge of tumor vessel expansion while undetectable levels have been reported in vessels of normal organs [22-23].

Functional studies have shown that TEM-1 knockout mice develop normally and exhibit normal wound healing, suggesting that TEM-1 is not required for neovascularization during fetal development or wound repair [24]. When colorectal cancer cells were implanted orthotopically in the abdominal sites of these knockout mice, the lack of TEM-1 expression correlated with a drastic reduction in tumor growth, invasion, and metastases as compared to parental animals. These results suggest that stromal and/or endothelial-associated cells expressing TEM-1 support tumor growth and invasion, perhaps via the interaction with cellular and ECM proteins associated in the microenvironment of the tissue of origin.

Molecular and cellular studies have found that TEM-1 is able to selectively bind to the ECM proteins fibronectin (FN) and collagen types I and IV (Col I, Col IV). Engineered cells expressing TEM-1 exhibit enhanced adhesion to FN as well as enhanced migration through tumor matrices containing this ECM protein [25]. In cells, TEM-1 has been shown to be directly involved in regulating cellular proliferation [26] and in a subset of cells this proliferation appears to involve the PDGFR-β pathway, a pathway reported to be highly associated with tumor stromal cell proliferation [6, 27].

Based on the important role of stroma in supporting tumor growth and the activity of TEM-1 on supporting tumor stromal cell functions, clinical studies using a humanized monoclonal antibody called ontuxizumab (MORAb-004) are currently being conducted to determine the safety and clinical activity of blocking TEM-1 function in patients with various cancer types [28]. To better support the goals of these and future studies, it is important to define the nature of disease expressing various levels of TEM-1 as a means to determine clinical outcome in patients potentially responding to ontuxizumab therapy. In light of the complex association of TEM-1 in tumor and tumor stroma, here we have defined a set of markers involved in the TEM-1 pathway. These markers include the hypoxia associated hypoxia-induced transcription factor 2 alpha (HIF2α) [29, 30] and carbonic anhydrase 9 (CAIX) [31] proteins; hypoxic regions within tumors have been described to express TEM-1 [30]. In addition, we assessed the expression of PDGFR-β, Col I, Col IV and FN, previously shown to be involved in the TEM-1 pathway/axis. Analysis of these proteins in tumors derived from colorectal cancer patients have identified patterns that further define the disease and are useful for studying TEM-1 expression and clinical response, as well as having potential use for determining patient prognosis once more mature clinical association data is gathered.

Here we describe the development of the TAPPS diagnostic marker panel that is useful in determining prognosis in colorectal carcinoma, especially Stage II disease and may be useful in aiding the detection and therapeutic direction of colorectal cancers.

## RESULTS

### MAb 9G5 Specificity

The specificity of the rat monoclonal anti-TEM-1 antibody, clone 9G5, was assessed by several techniques, including fluorescence activated cell sorting (FACS) analysis. As shown in Fig. S1, MAb 9G5 specifically recognized TEM-1 on aortic smooth muscle cells (AoSMC) with little to no binding to human umbilical vein endothelial cells (HUVEC), as expected. Further, fluorescently labeled secondary antibody (goat anti-rat IgG) showed little to no background. These data and other specificity studies (not shown) indicate that MAb 9G5 is specific for human TEM-1.

### Total Expression Analysis

**Fig 1:**
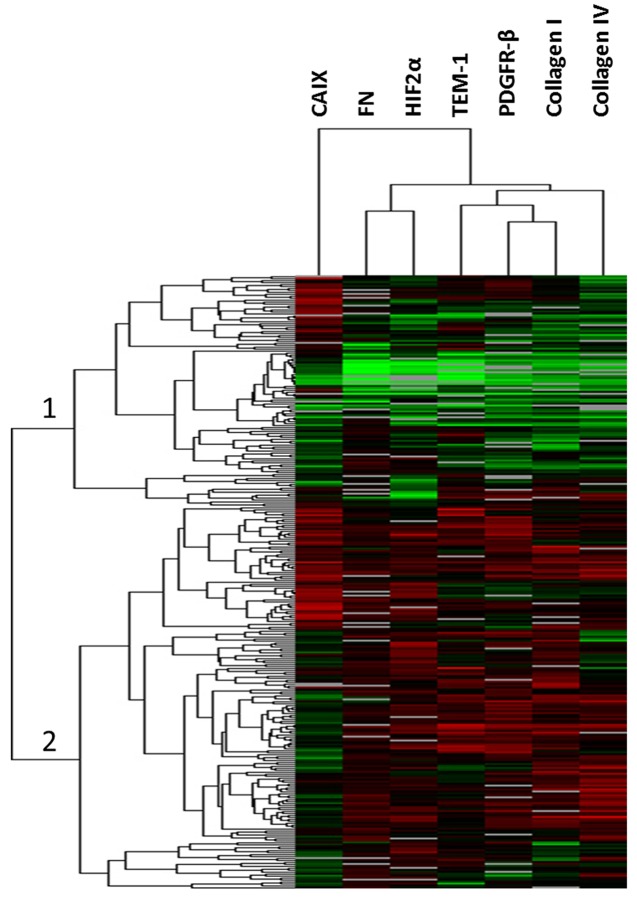
Unsupervised hierarchical clustering Total expression AQUA scores were clustered across all patients resulting in two main clusters (1 and 2, indicated) of patients. Each bar represents an individual patient for each indicated biomarker (top) with increasing red color (black to red) indicating increased expression and decreasing green color (black to green) indicating decreasing expression. Gray bars indicate missing data point.

Since all TEM-1 associated biomarkers [TEM-1, HIF2α, CAIX, PDGFR-β, FN, Col I and Col IV] have shown putative expression in both tumor and stromal cellular compartments, we first examined total tissue level expression on the Colorectal Cancer (CRC) cohort, described in Table 1. Unsupervised hierarchical clustering revealed two main patient clusters (Fig. 1). In cluster 1, all patients expressed relatively low levels of all proteins, while in cluster 2 all patient samples expressed relatively high levels of all proteins except CAIX. For biomarker (“array”) clustering, TEM-1 clustered most closely with PDGFR-β, Col I and Col IV. This also bore out in bivariate correlation analysis with significant (p<0.001; α=0.001 based on Bonferroni correction for multiple comparisons) Spearman's Rho correlation coefficients of 0.62, 0.43, and 0.31, respectively. A complete bivariate correlation analysis is provided in Table 2. CAIX did not cluster with any of the other biomarkers tested and thus is the least related. When we examined survival outcomes as a function of total expression, while all markers demonstrated an optimal cut-point on the training set for predicted survival (p<0.10), none of these cut-points held up in the validation set (Fig. S2).

**Table 1:** Cohort Summary Demographic summary of available clinical data for training and validation sets with Cox Proportional Hazards modeling based on 5-year disease specific survival. Provided are number (N) and group percentages for each clinical variable, hazard ratios (HR) and 95% confidence intervals (95%CI) and p-values. HR is based on comparison to first category. Also provided are Chi-square p-values for comparison of the relative proportion of cases in each group between the training set and validation set. No significant difference in case proportion was observed

	Training Set (n=330)			Validation Set (n=164)			χ^2^p
Variable	N*(%)	HR^(95%CI)	P^	N (%)	HR (95%CI)	P	
Duke's Stage I II III IV	74 (23) 84 (27) 130 (41) 29 (9)	2.7 (1.3-5.5) 5.8 (3.0-11.2) 3.0 (1.2-7.2)	0.008 <0.001 0.02	31 (21) 42 (28) 55 (37) 21 (14)	1.8 (0.7-4.6) 3.3 (1.4-8.1) 1.3 (0.4-4.1)	0.24 0.007 0.71	0.37
Grade Well Moderate Poor	103 (38) 140 (52) 27 (10)	1.2 (0.8-1.8) 1.1 (0.6-2.3)	0.40 0.70	60 (46) 55 (42) 15 (12)	1.6 (0.8-2.9) 2.1 (0.9-5.0)	0.15 0.10	0.20
Sex Female Male	176 (53) 154 (47)	1.4 (1.0-2.0)	0.05	98 (60) 66 (40)	1.4 (0.8-2.3)	0.2	0.18
Age 40-50 50-60 60-70 >70	16 (5) 68 (21) 107 (32) 139 (42)	0.6 (0.3-1.4) 0.6 (0.3 − 1.4) 0.8 (0.3-1.7)	0.23 0.28 0.49	9 (6) 35 (21) 49 (30) 71 (43)	0.5 (0.2-1.2) 0.4 (0.2-1.1) 0.5 (0.2-1.5)	0.12 0.09 0.30	0.89

* Clinical data not available on all cases; percentages based on total number in category

^ Hazard ratios (HR), 95% Confidence Intervals (95%CI), and P-Values (P) based on 5-year disease-specific Cox Proportional Hazards modeling

**Table 2:** Biomarker correlation summary For each biomarker pairing, Spearman Rho Correlation Coefficients and p-values are provided. Black boxes indicate non-applicable analysis same marker correlations.

		TEM-1	PDGFR	Fibronectin	CAIX	HIF2α	Collagen I	Collagen IV
TEM-1	Correlation1		.624**	.293**	.168**	.284**	.431**	.310**
	Sig. (2-tailed)		<0.001	<0.001	0.004	<0.001	<0.001	<0.001
PDGFR	Correlation1	.624**		.333**	.240**	.449**	.652**	.497**
	Sig. (2-tailed)	<0.001		<0.001	<0.001	<0.001	<0.001	<0.001
Fibronectin	Correlation1	.293**	.333**		0.058	.543**	.469**	.449**
	Sig. (2-tailed)	<0.001	<0.001		0.33	<0.001	<0.001	<0.001
CAIX	Correlation1	.168**	.240**	0.058		.119*	.127*	0.061
	Sig. (2-tailed)	0.004	<0.001	0.33		0.04	0.03	0.3
HIF2α	Correlation1	.284**	.449**	.543**	.119*		.465**	.302**
	Sig. (2-tailed)	<0.001	<0.001	<0.001	0.04		<0.001	<0.001
Collagen I	Correlation1	.431**	.652**	.469**	.127*	.465**		.644**
	Sig. (2-tailed)	<0.001	<0.001	<0.001	0.03	<0.001		<0.001
Collagen IV	Correlation1	.310**	.497**	.449**	0.061	.302**	.644**	
	Sig. (2-tailed)	<0.001	<0.001	<0.001	0.3	<0.001	<0.001	

1 Correlations were classified as follows: ** indicating highly significant (P<0.01) and * indicating significant (0.05 > P > 0.01).

### Architectural Context-specific expression of biomarkers

**Fig 2:**
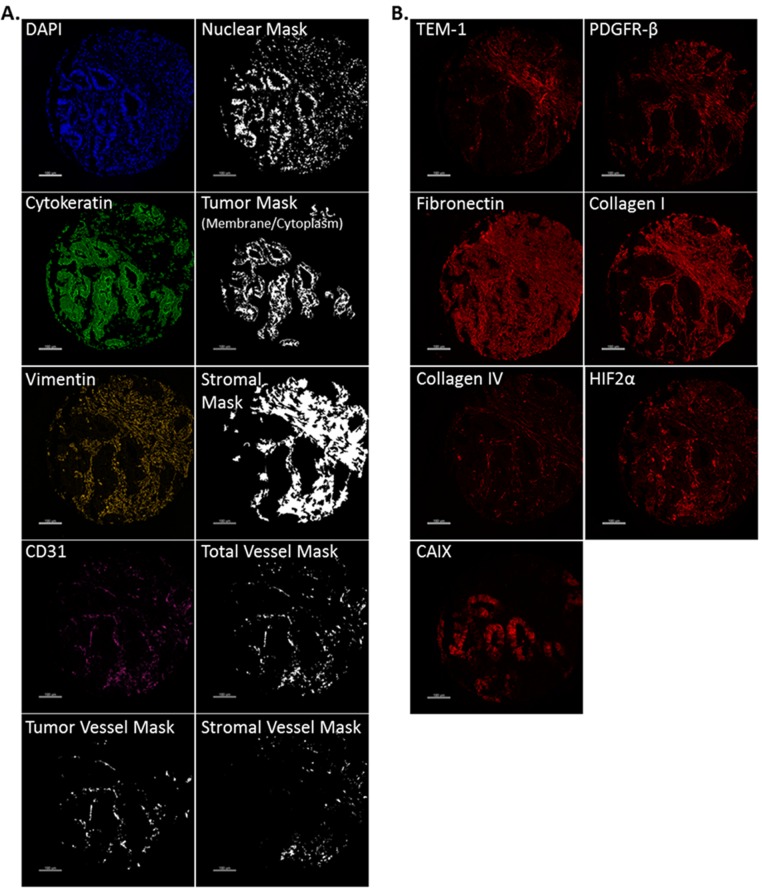
Biological compartment masking and biomarker example image panel (A) Representative image examples from each fluorescence channel used to generate architectural compartment masks during AQUA analysis. DAPI was used to generate a nuclear mask, cytokeratin (FITC) to identify tumor membrane/cytoplasm, vimentin (Cy3) to identify stroma, CD31 (Cy7) was used to identify vasculature, and in conjunction with cytokeratin to determine tumor vasculature and with vimentin to identify stromal vasculature. (B) Representative image examples of the TEM-1 associated biomarker panel (Cy5).

Since each of these markers potentially show architecturally-defined cellular expression, we developed compartment specific “masks” that specifically identify and isolate tumor, stroma, stromal-specific vasculature and tumor-specific vasculature. In brief, each TEM-1 associated biomarker was combined with DAPI to identify nuclei, cytokeratin (CK) to identify tumor membrane/cytoplasm, vimentin to identify stroma and CD31 to identify vasculature. We then used advanced image analysis algorithms (AQUA technology) to generate a binary image identifying each pixel as either included or excluded from each compartment of interest. Fig. 2A provides example images of each compartment-specific marker and the respective compartments used in this study. Fig. 2B provides an example of the staining localization seen with each of the TEM-1 associated biomarkers analyzed on the same given sample. AQUA scores for each biomarker were generated for the 4 biological compartments of interest: tumor (membrane/cytoplasm), tumor vessel, stroma (stroma without contributing vasculature) and stromal vessel.

**Fig 3:**
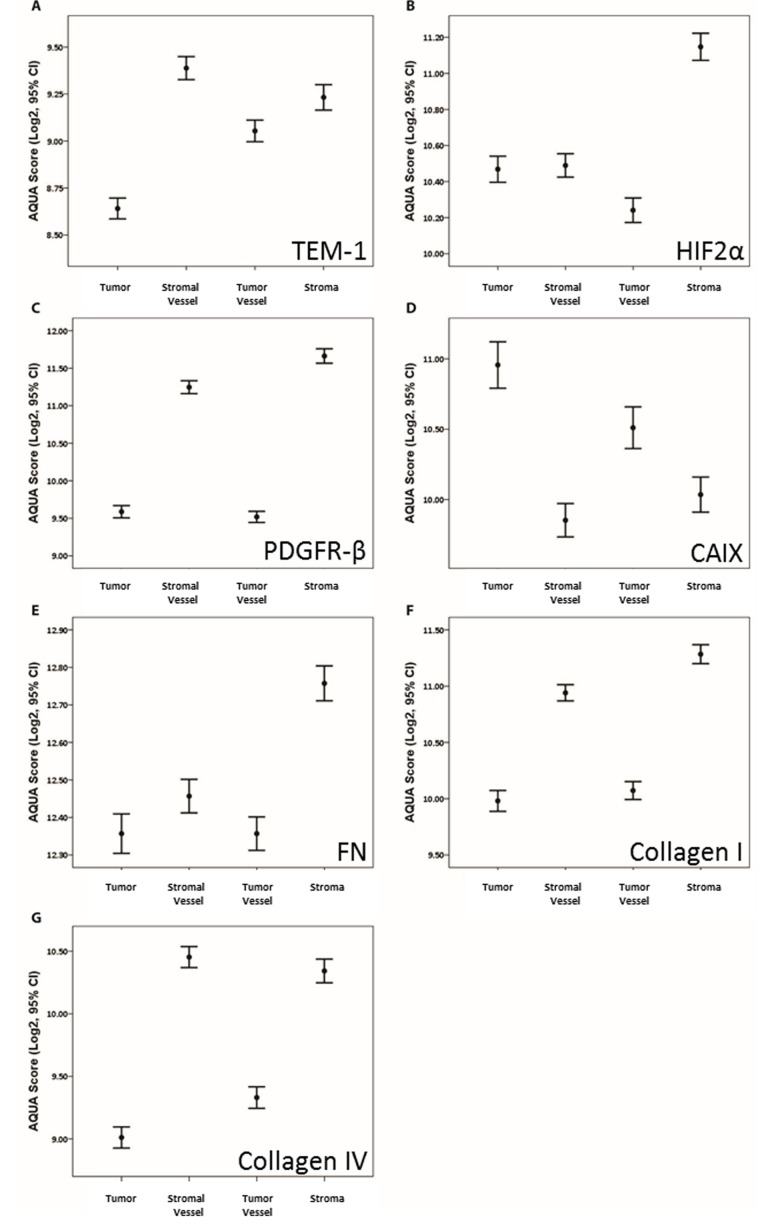
Means analysis of AQUA scores by architectural compartment for each biomarker Means plots with AQUA score means and ±95% confidence intervals of the mean (error bars) for each indicated architectural compartment for (A) TEM-1; (B) HIF2α; (C) PDGFR-β; (D) CAIX; (E) FN; (F) Col I; and (G) Col IV.

Means analysis showed significant differential expression by compartment (Fig. 3). All markers, except for CAIX, showed significantly higher expression in stroma and/or vasculature compared to tumor. Conversely, CAIX showed higher level expression in tumor and tumor vessel compared to stromal components. Expression as a function of available clinical variables (i.e. Stage and Grade) showed only minimal association (data not shown). Specifically, PDGFR-β and Col IV showed significantly higher expression in males compared to females; and stromal FN and Col I showed significantly higher expression in advanced staged disease.

### Univariate Survival Analysis

**Fig 4:**
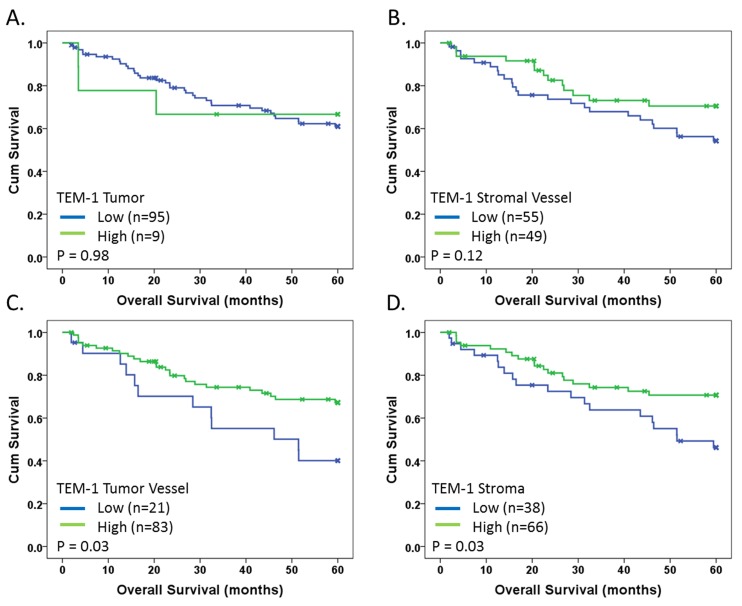
Kaplan-Meier survival analysis of TEM-1 Kaplan-Meier 5-year disease-specific survival analysis with indicated p-values for TEM-1 in indicated architectural compartments: (A) Tumor; (B) Stromal Vessel; (C) Tumor Vessel; and (D) Stroma. Blue and green lines indicate low and high level expression, respectively. X-marks indicate censored cases. Cut-points were determined based on optimal cut-point analysis.

Optimal Kaplan-Meier survival cut-points were defined within continuous AQUA score data using X-Tile analysis (see Materials and Methods) on the training set (Table 1) and subsequently applied to the validation set. The cut-points do not necessarily reflect positive/negative cut-points but rather, expression thresholds as defined as a function of survival. The individual biomarker was considered to validate if significance (p<0.05) was reached in both the training set and validation set. Five-year disease-free survival data for each individual marker/compartment combination is summarized in Table S1. Although all markers showed a trend for association with survival (Training p<0.20), stromal TEM-1 expression as well as stromal vessel Col I and HIF2α were the only biomarkers to validate in the univariate setting. High tumor vessel and stromal TEM-1 expression, as well as stromal vessel HIF2α, associated with increased five-year disease specific survival (Table S1), while high stromal vessel Col I expression associated with decreased five-year survival (Table S1). Kaplan-Meier survival analysis of the validation set for TEM-1 expression in all compartments is shown in Fig. 4 demonstrating the differential survival benefit and thus justification for quantifying biomarker expression in distinct cellular or architectural compartments.

### Generation of TAPPS score model

Because of the association of these markers (Fig. 1) and the putative impact these markers together may have on both survival as well as potential response to TEM-1 targeted therapy via ontuxizumab, we combined markers in a multivariate Cox Proportional Hazards model using the univariate cut-points. The criterion for initial entry into the model was statistical significance at the 20% level in both the training set and validation set (biomarkers highlighted in bold in Table S1; n = 13). Using backward elimination modeling based on Wald statistics on the training set, the starting model of 13 biomarkers was refined down to 5 biomarkers (Table 3): TEM-1 stroma, TEM-1 tumor vessel, HIF2α stromal vessel, Col IV tumor, and FN stroma. This overall model was highly significant (p<0.001).

**Table 3:** Summary of TAPPS model development Results from multivariate Cox Proportional Hazards modeling for the full model (all pre-qualified markers entered into model) and the minimal model (markers rationally chosen from full model) with indicated Model P values. Provided are the marker in the model, multivariate HR, 95%CI, p-values and model coefficient (if applicable). Cox Proportional Hazard modeling results based on continuous TAPPS scores for both training set and validation set are provided for the full and minimal models.

Model	Marker	HR	95%CI	P-value	Coefficient
Full (Individual Markers)	TEM1 Stroma	0.41	0.24-0.69	0.001	−0.89
	TEM1 Tumor Vessel	3.27	1.35-7.95	0.009	1.19
	HIF2α Stromal Vessel	0.47	0.25-0.88	0.02	−0.76
	Collagen IV Tumor	1.86	1.08-3.21	0.03	0.62
	FN Stroma	2.29	1.23-3.21	0.009	0.83
Full (TAPPS Score)	TAPPS (Training)n=330	1.76	1.44-2.15	<0.001	NA
	TAPPS (Validation)n=164	1.38	1.02-1.88	0.04	NA
	TAPPS (w/Clinical)n=494	1.66	1.37-2.03	<0.001	NA
	TAPPS (Stage II)n=126	1.75	1.22-2.52	0.002	NA
	TAPPS (Stage III/IV)n=235	1.40	1.14-1.73	0.001	NA
Minimal (Individual Markers)	TEM-1 Stroma	0.58	0.36-0.93	0.03	−0.55
	Collagen IV Tumor	1.90	1.12-3.25	0.02	0.64
	FN Stroma	2.18	1.18-4.05	0.01	0.78
Minimal (TAPPS Score)	mTAPPS (Training)n=330	2.72	1.82-4.08	<0.001	NA
	mTAPPS (Validation)n=164	2.66	1.26-5.62	0.01	NA
	mTAPPS (w/Clinical)n=494	2.37	1.48-3.81	<0.001	NA
	mTAPPS (Stage II)n=126	3.99	1.71-9.29	0.001	NA
	mTAPPS (Stage III/IV)n=234	1.94	1.27-2.98	0.002	NA

Taking the model coefficients for each marker (Table 3) we derived an equation to provide an overall risk score. We termed this the TAPPS (TEM-1 Associated Pathway Prognostic Signature) score and it is defined as:

TAPPS = (TEM-1 Stroma _(0/1)_ * −0.89) + (TEM-1 Tumor Vessel _(0/1)_ + 1.19) + (HIF2α Stromal Vessel _(0/1)_ * −0.76) + (Col IV Tumor _(0/1)_ * 0.62) + (FN Stroma _(0/1)_ * 0.83)

There was sufficient marker data for each of these five markers to generate a TAPPS score on 256 patients. The resultant TAPPS score had a range of −0.89 to 3.88 with a median score of 1.13. As a continuous variable on the training set, as expected, the TAPPS score significantly associated with decreased survival [HR = 1.76 (95% CI: 1.44-2.15); p < 0.001; Table 3] and was applied to the validation set with significance [HR = 1.38 (95%CI: 1.02 − 1.88); p = 0.04; Table 3]. Importantly, the TAPPS score was independent and provided significant added prognostic value [HR = 1.66 (95%CI: 1.37-2.03); p<0.001; LR-χ^2^ = 67.2] when put into an already highly significant model with known clinical variables (Table 1; LR-χ^2^ = 49.7).

**Fig 5:**
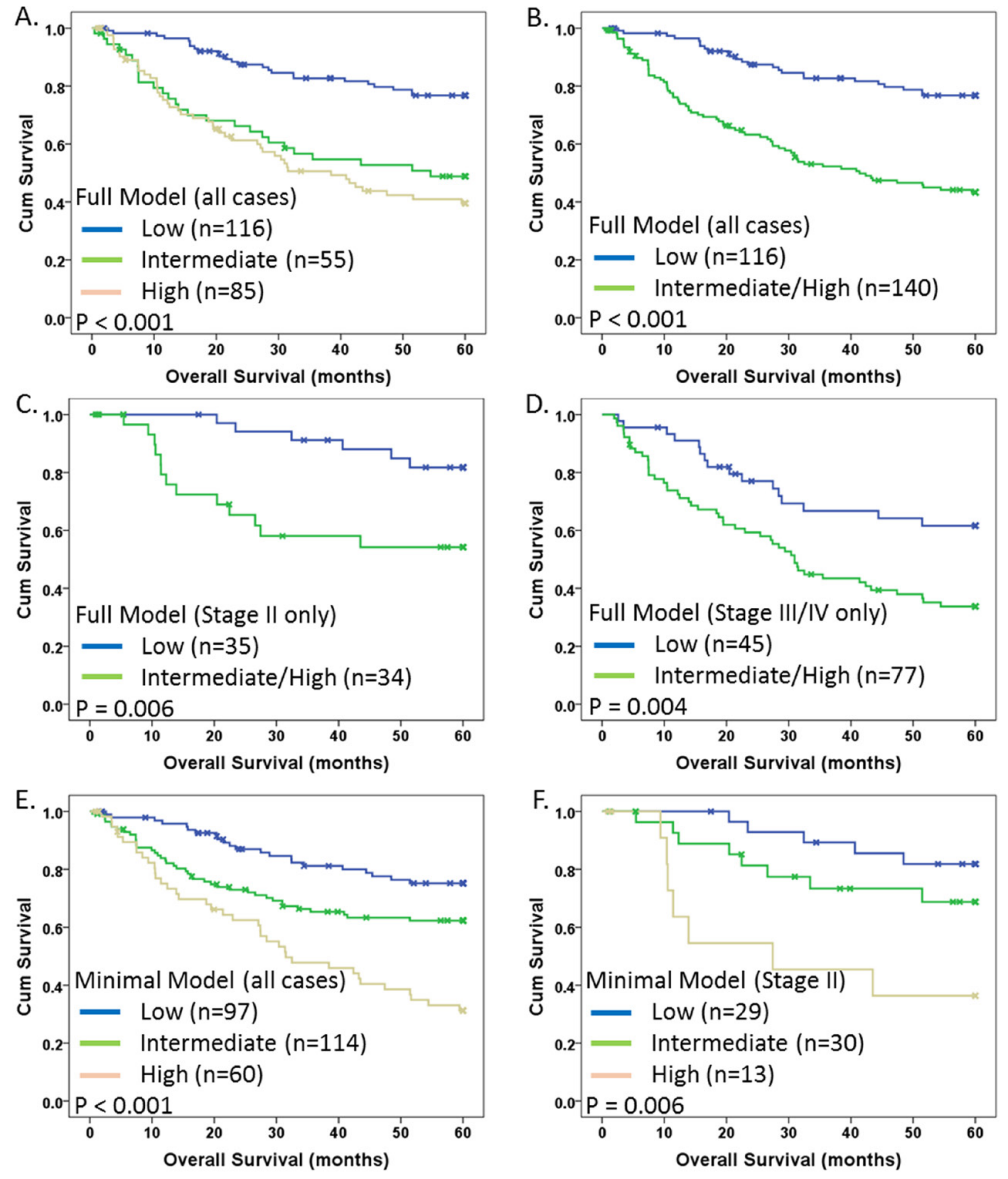
Kaplan-Meier survival analysis of TAPPS modeling Kaplan-Meier 5-year disease specific survival analysis with indicated p-values for TAPPS risk groups for indicated patient groups: (A) Full model with all cases using 3 risk groups; (B) Full model with all cases using 2 risk groups (high and intermediate from (A) combined into single high risk group); (C) Full model with only Stage II cases; (D) Full model with only Stage III/IV cases; (E) Minimal model (mTAPPS) with all cases (3 risk groups); and (F) mTAPPS with Stage II only (3 risk groups). (A,E,F): Blue, green, and beige lines indicate low, intermediate and high risk groups respectively. (B,C,D): Blue and green lines represent low and high risk groups, respectively. X-marks indicate censored cases.

In order for the TAPPS score to be most useful as a diagnostic in the clinical setting, risk groups (similar to groups of patients classified by the OncotypeDx score) should be established as a function of the continuous risk score to allow for classification of patients. Therefore, we divided the TAPPS score into three groups based on distribution analysis, representing putatively low risk (TAPPS < 0.3), intermediate risk (TAPPS 0.3 – 1.86), and high risk (TAPPS > 1.86). We chose to perform this analysis, and subsequent analyses on all cases, since we had previously validated the TAPPS score via training/validation set analysis. When survival was examined by Kaplan-Meier, although highly significant (p<0.001), there was no substantial difference between intermediate and high risk groups (Fig. 5A). Therefore, we combined the intermediate and high risk groups to form an intermediate/high risk group (Fig. 5B) and retained significance (p<0.001), as expected.

An important clinical question in the treatment of CRC is whether to provide chemotherapy for Stage II patients and thus there is value in a biomarker signature that could differentiate Stage II patients based on overall risk. We therefore examined the association of the TAPPS risk groups in Stage II patients only and observed a highly significant association with survival (p=0.006; Fig. 5C) with a HR = 3.5 (95%CI: 1.3-9.4; p = 0.01). A significant association in Stage III/IV patients (Fig. 5D) was also observed.

Lastly, for simplicity in potentially moving this biomarker signature into the clinical and/or companion diagnostic setting, we sought to reduce the number of markers in the model to ideally 3 or less to minimize assay complexity and sample (tissue slide) requirements, often a limiting factor. Rationally, removing the vessel markers would eliminate the requirement for the CD31 (vasculature) compartment. Therefore, we tested a model with only TEM-1 stroma, Col IV tumor and FN stroma. Although this minimal model (mTAPPS; n=271 total) did not provide as much prognostic value (LR-χ^2^ = 32.2 for the minimal versus LR-χ^2^ = 37.2 for the full model), it was nonetheless highly significant [HR = 2.7 (95%CI: 1.9-3.8); p<0.001) even when evaluated in the presence of clinical variables [HR = 2.37 (95%CI: 1.48-3.81); p<0.001]. Further validation of these models is required prior to clinical application and is discussed below.

## DISCUSSION

Here we provide evidence that demonstrates expression of tumor and tumor stromal markers, in architectural context, in colorectal cancer defines populations with distinctively different outcomes thereby providing valuable prognostic information. The TEM-1 pathway, which is involved in supporting and promoting tumor growth via direct and indirect mechanisms, was used to determine whether or not its expression pattern in CRC could define clinical outcomes. Here we have shown that TEM-1 and a subset of its associated pathway partners can indeed predict outcomes of patients with CRC. The TAPPS score described herein could be useful in clinical practice using standard of care therapeutic regimens to aid in predicting patient chemotherapeutic benefit, especially in Stage II patients. Moreover, TEM-1 targeted therapies may benefit from the TAPPS scoring paradigm as it assesses, with respect to disease outcome, not only TEM-1 but also TEM-1 associated proteins. We hypothesize that results integration of multiple biomarkers will provide greater predictive information compared to assessment of a single marker, as we show here relative to prognosis. This information may further enable the development of clinical trials to determine the ability of improving clinical outcomes in patients with poor prognosis by stratifying patients with high versus low TAPPS scores to determine whether or not a therapeutic regimen can improve outcomes in these backgrounds.

A limitation of the present study is that it was performed on a single, older cohort with little to no treatment information. As such we approached this study as a pure discovery translational study looking to examine potential associations of known markers with clinical variables such as stage and grade as well as with overall survival. Although the cohort was large enough to reasonably divide into training and validation sets, these findings remain to be validated in a completely independent cohort. However, the results are compelling based on the level of significance and independence of the TAPPS score from clinical variables, specifically stage of disease. Importantly, while stage II disease is generally a good prognosis, at least relative to late stage disease, the TAPPS score was clearly able to discriminate prognostic sub-groups within this population. The present study was also limited by having only a single core from each patient on the tissue microarray. Future validation studies will need to examine multiple cores and/or whole tissue sections to determine the effects of expression heterogeneity on the model. Such validation studies are in the planning stages.

There is a critical clinical need to identify CRC patients at lower risk of progression, especially in Stage II patients where although surgical resection results in 75-80% 5-year survival, there is a substantial number of patients that recur. Thus, development of risk assessment algorithms, such as the TAPPS score described here, is critical to meet this need and provide the oncologist and pathologist the means to determine which patients will most likely benefit, and perhaps even more importantly, not benefit from toxic chemotherapy. Genomic Health has developed the OncotypeDx test for CRC patients specifically for patients with Stage II or Stage III A/B disease. Studies should be undertaken to compare the TAPPS score with the OncotypeDx CRC test especially since the latter contains several stromal markers not present in the TAPPS score. The potential additive prognostic and/or predictive power of these two approaches should be evaluated.

Furthermore, given that the TEM-1 pathway has been found to be ubiquitously expressed in virtually all cancers tested to date, analysis of this pathway, and more specifically the TAPPS score, in other cancer types may be useful for defining patient populations across a number of cancer indications and provide a guide for physicians to direct therapy for patients. Studies in other cancer types are ongoing.

Only a few studies in humans [10, 15-20] and several in mice [24, 32, 33], have been conducted relative to the apparent prognostic effect of TEM-1 measurements, by either RNA expression or IHC. A small preliminary study in 31 CRC patients [10] suggested that TEM-1 expression correlates with advanced disease and thus potentially decreased survival. Importantly, this study did not examine time to event (prognosis) directly, but, rather, association with stage of disease. Total TEM-1 expression was also not prognostic in the present cohort (Fig. S2), consistent with previous observations. However, the expression of TEM-1 in an architecturally-specific context, in particular tumor vessels and stroma, was mildly prognostic (p=0.03; Fig. 4) in univariate analysis. This apparent disparity with literature may be reflective of both the power of the present study as well as the objective and localized expression analysis of TEM-1 that may be more reflective of TEM-1 biology.

It is also important to note however that prognosis flipped in multivariate modeling for TEM-1 tumor vessel expression when taken in context with other markers. These data might suggest that TEM-1 prognostic value is tied to the expression of its associated pathway markers and hence lends support to the fact that assessment of multiple markers in the TEM-1 pathway is required to achieve accurate prognosis.

Taken together, the results of this study identify a set of markers whose architecturally-specific quantitative expression, when integrated together into a single algorithm (TAPPS), provides significant prognostic value for the prediction of survival in CRC patients. This work represents a discovery translational study that requires further validation in independent cohorts, specifically clinical trial cohorts with controlled treatment settings, to determine the value of the TAPPS score at predicting response to chemotherapy, especially in Stage II and/or Stage III A/B patients. Further validation of the TAPPS score would justify clinical development and commercialization to provide physicians with testing options for determining how best to treat their patients.

## MATERIALS AND METHODS

### Cohort Description

Tissue microarrays (TMAs) originally containing a total of 599 primary colorectal carcinomas (CRCs) from formalin fixed, paraffin-embedded tumor samples obtained at Yale University-New Haven Hospital (New Haven, CT) from 1970-1981, were constructed at the Yale University Tissue Microarray Facility (New Haven, CT) as described in detail elsewhere (34, 35). Of 599 cases, 494 had both biomarker data and clinical data. The cohort was split into training (67%) and validation (33%) sets based on sequential enrollment from diagnosis date (2 cases to the training set, and 1 case to the validation set).

Clinical variables available for this cohort included Duke's Staging, histological grade, sex, and age. Chi-square analysis showed no significant differences in the proportion of clinical variables between training and validation sets (Table 1). Median disease-specific follow-up was 24 months with a median age of 68 years. Cox Proportional Hazard modeling based on five-year disease-specific survival for each clinical variable showed the expected decreases in survival for advanced stage and males, but no significant differences were observed for histological grade or age (Table 1). Although Stage IV and poor grade patients appear to perform better, this is likely due to differences in observational power based on substantially reduced case numbers.

### Immunofluorescence Staining

Immunofluorescence staining for AQUA technology has been described in detail previously [36, 37]. A summary of antibody reagents used is provided in Table S2. In brief, pre-cut paraffin-coated tissue microarray slides, one for each protein biomarker, were de-paraffinized. Antigen retrieval for slides to be stained with TEM-1 and CAIX was performed using a Decloaking Chamber with a 10X DIVA buffer, pH 6.2 (Biocare Medical, Concord, CA): slides were incubated for 15 minutes inside the chamber where pressurized incubation reached a maximum of 125°C at 15-20 PSI for 30 seconds followed by cool down for 15 minutes down to 95°C. Antigen retrieval for slides to be stained with PDGFRβ, FN, HIF2α, Col I and Col IV were done in a PT Module (Labvision, Fremont, CA) with Tris/EDTA buffer, pH 9. Differential antigen retrieval conditions were determined empirically based on optimal staining specificity and dynamic range of expression with retrieval in Tris/EDTA pH 9 being the starting condition.

Staining for all markers was performed on a LabVision Autostainer (Labvision) according to previously described protocols. All steps were carried out at room temperature and each step was separated by three TBS-Tween rinses, unless otherwise specified. Antibodies were diluted in Davinci Green (Biocare Medical) unless otherwise specified. Driven by antibody species considerations, different anti-CD31 antibodies (mouse or rabbit; see Table S2) were used in combination with anti-target antibodies in conjunction with species specific secondary reagents (Table S2). The general staining workflow proceeded as follows: 1) CD31 detection; 2) Target and vimentin detection; and 3) Cytokeratin detection. Both CD31 detection and target detection required signal amplification mediated by horseradish peroxidase (HRP), thus a dual-amplification approach was taken by which CD31-associated HRP enzymatic activity was quenched by 100 mM Benzhydrazide (Sigma Aldrich, St. Louis, MO) plus 50 mM hydrogen peroxide (Sigma Aldrich) prior to target and vimentin detection.

For CD31 detection, slides were incubated for one hour at room temperature with mouse anti-CD31 or rabbit anti-CD31, followed by Envision Plus (mouse or rabbit depending on species of target antibody) for 30 minutes. Slides were then incubated with biotinylated tyramide (TSA plus Biotin system, Perkin Elmer, Waltham, MA) for 10 minutes, followed by two incubations of benzoic hydrazide and hydrogen peroxide for 8 minutes and 7 minutes. Slides were incubated with Alexa Fluor 750 streptavidin for 30 minutes for detection of CD31 in the 750 nm (Cy7) imaging channel.

For target and vimentin detection, primary antibodies and anti-vimentin antibody were incubated for one hour followed by a 30 minute incubation with Immpress reagents (rat, rabbit, or mouse depending on species of target antibody), then 30 minutes with goat anti-chicken Alexa Fluor 555. Tissue samples were then blocked with mouse IgG block (BioCare Medical) followed by incubation with Alexa488 Pan Cytokeratin for 30 minutes at room temperature for Cytokeratin detection. Slides were then incubated with the Cy5 tyramide amplification system (Perkin Elmer) for 10 minutes as the final step for target detection, then mounted with Prolong anti-fade with DAPI (Invitrogen, Carlsbad, CA) for the identification of nuclei within each sample. To control for potential HRP/TSA background, we have demonstrated (unpublished results) through no primary and IgG antibody controls that there is no significant background fluorescence intensity contribution from HRP secondary and/or TSA reagents on CRC tissues.

### Imaging and Image Analysis

Relative protein concentration within subcellular or architectural compartments can be measured with a high degree of precision using the AQUA analysis system, as described in detail previously [36, 37]. In brief, high resolution, 12 bit (resulting in 4096 discrete intensity values per pixel of an acquired image) digital images of the cytokeratin with FITC, vimentin with Cy3, nuclear staining with DAPI, biomarker panel staining with Cy5 and CD31 with Cy7 were captured and saved for every histospot on the array using the PM2000 epi-fluoresence microscopy system (HistoRx, Inc., New Haven, CT). Prior to statistical analysis, images are reviewed for quality (e.g. poor tissue, saturation, focus and other artifacts) and signal intensity of all compartment channels. The pan-cytokeratin signal was used to create an epithelial mask to distinguish regions of epithelial tissue from stromal elements within both the normal and tumor samples. Vimentin was used to create a total stroma specific mask and CD31 was used to create a vasculature specific mask. Using the combination of these masks we were able to generate tumor vasculature (termed “Tumor Vessel”), stromal vasculature (termed “Stromal Vessel”) and pure stroma (Total Stroma excluding Stroma vasculatures termed “Stroma”) compartments.

### Assay Development

Quantitative immunofluorescence assays were developed as previously described (38). In brief, antibody tissue staining was judged qualitatively for specificity of staining on a multi-tumor TMA test cohort [38] across a range of primary antibody dilutions. The antibody concentration with the optimal dynamic range was chosen as optimal. This approach is critical since judgment by eye is biased towards higher antibody concentrations. Better quantitative comparisons, with clinical features and outcome, are obtained by maximizing the dynamic range of the assay such that as many patients in the population are placed within the linear range of the assay.

Further, the TEM-1 assay was assessed for performance and precision (precision assessment on the other assays is in progress) by running the assay on three independent staining days (three slides each) and imaged on three independent instruments by two independent operators on the same multi-tumor TMA cohort used for assay development. Overall performance was assessed by linear regression analysis (Pearson's R and slope) while assay precision was assessed by examination of %CV. Established acceptance criteria performance and precision are defined as a Pearson's R > 0.9, slope 0.8 – 1.2 and %CV < 15%. These criteria were determined empirically for the AQUA platform as well as based on industry standards for immunoassay reproducibility by examination of quantitative AQUA scores. The results of TEM-1 performance and precision are provided in Table S3. The TEM-1 AQUA assay demonstrated excellent precision with an average Pearson's R of 0.96 and average slope of 0.998, as well as performance with a day-to-day %CV of 4.1%. Additionally, as a measure of assay specificity, we compared quantitative results of this 9G5 TEM-1 assay with a commercially available anti-TEM-1 rabbit polyclonal antibody (Sigma Aldrich, St. Louis, MO) and observed strongly correlative results (Pearson's R = 0.79).

### Statistical Analysis

Unsupervised hierarchical clustering was performed using the Multiple Experiment Viewer of the TM4 Microarray Software Suite [39]. Clustering was performed using average linkage clustering by Pearson's uncentered correlation. All statistical analyses were conducted using SPSS v17 or later (SPSS Inc., Chicago, IL). AQUA scores for all biomarkers showed a skewed distribution and therefore the log base 2 transformed scores were used for subsequent parametric analyses (i.e. means comparisons). However, to provide linear comparisons, raw AQUA score data is sometimes reported. Differences in mean scores between clinical features were assessed by general linear modeling based on one-way ANOVA. Optimal cut-point analysis for biomarkers on the training set with respect to 5-year disease specific survival was performed using X-Tile™ [40]. Cox proportional hazards modeling was performed in the multivariate setting using backwards elimination by Wald Statistics for determining the optimal analytical model. Log-likelihood chi-square ratios (LR-χ^2^) were used to compare models. All survival analysis was based on five-year disease-specific survival. Kaplan-Meier survival analysis was compared using log-rank statistics. For survival analysis, although multiple comparisons were made for each marker (i.e. optimal cut-point analysis), p-values were not corrected since a training/validation approach was chosen, and markers were only considered to be significant if p<0.05 for both training and validation sets.

## SUPPLEMENTARY FIGURES AND TABLES


