# Supplementary material for: Influence of tumor microenvironment on prognosis in colorectal cancer: Tissue architecture-dependent signature of endosialin (TEM-1) and associated proteins
Source: Oncotarget. 2014 Jun 16;5(12):3983–95. doi: 10.18632/oncotarget.2108 (PMC4147300; doi:10.18632/oncotarget.2108)
Supplement: Supplementary file 1 [file oncotarget-05-3983-s001.pdf]

Influence of Tumor Microenvironment on Prognosis in Colorectal Cancer: Tissue architecture-dependent signature of endosialin (TEM-1) and associated proteins

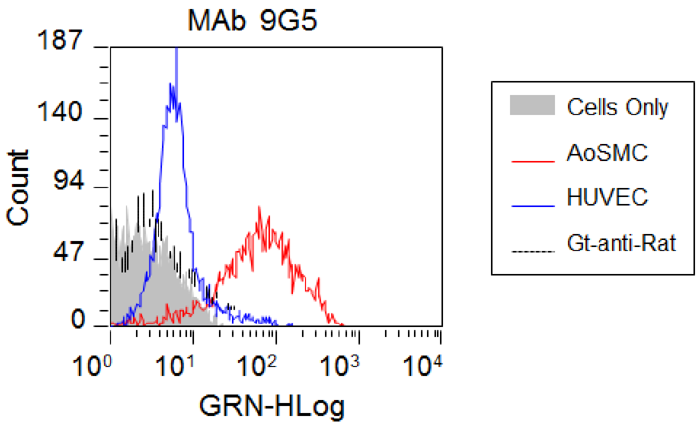

Histogram #	Geometric Mean
1	2.5
2	57.4
3	6.5
4	3.5

Fig. S1: Fluorescence activated cell sorting using anti-TEM-1 clone 9G5. Cell sorting was performed on aortic smooth muscle cells (AoSMC) and human umbilical vein endothelial cells (HUVEC). Clone 9G5 (rat IgG1) showed strong positive staining on AoSMC and very weak staining on HUVEC. Second antibody (goat anti-rat IgG) was completely negative.

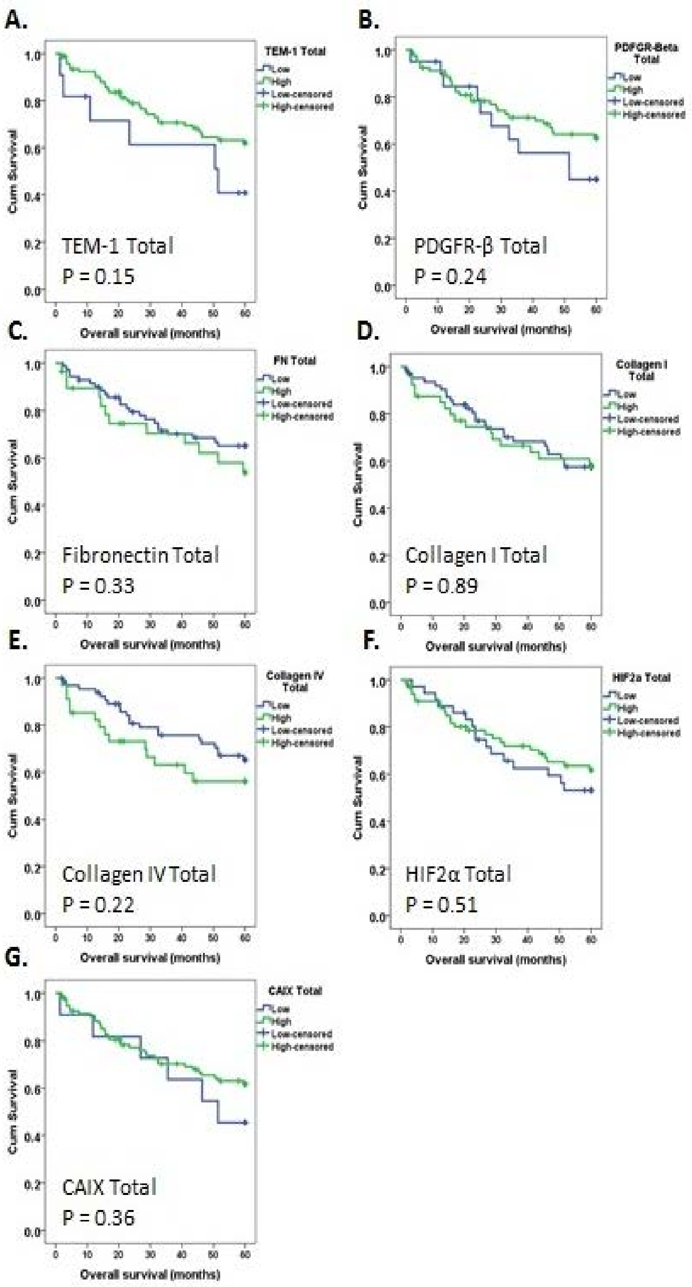

Fig. S2: Kaplan-Meier survival analysis for total expression of each biomarker. Kaplan-Meier 5-year disease-specific survival analysis with indicated p-values for all markers (total expression): (A) TEM-1; (B) PDGFR-β; (C) FN; (D) Col I; (E) Col IV, (F) HIF2α; and (G) CAIX. Blue and green lines indicate low and high level expression, respectively. X-marks indicate censored cases. Cut-points were determined based on optimal cut-point analysis.

Table S1: Summary of Univariate Survival Analysis. Univariate survival data is presented based on optimal cut-point analysis for each biomarker in each compartment. Provided are the determined optimal cut-points and % of high level expressing cases (% High), p-value for the Training Set (Training P), as well as hazard ratio (HR), 95% confidence intervals (95%CI) and Validation Set p-values (Validation P) based on 5-year disease-specific Cox Proportional Hazards modeling. Markers that are significant individual biomarkers for survival prediction are indicated as italicized bold while markers that were used in multivariate modeling resulting the TAPPS model are indicated in bold.

Marker	Optimal Cut (% High)	Training P	Validation HR (95%CI)	Validation P
TEM-1 Tumor	618.30 (10.1)	0.10	0.99 (0.30-3.21)	0.98
**TEM-1 Stromal Vessel**	**635.84 (50.7)**	**0.12**	**0.59 (0.30-1.16)**	**0.12**
**TEM-1 Tumor Vessel**	**364.27 (85.0)**	**0.19**	**0.47 (0.24-0.94)**	**0.03**
TEM-1 Stroma	***473.58 (65.2)***	***0.012***	***0.49 (0.26-0.94)***	***0.03***
**PDGFR-β Tumor**	**1407.43 (13.1)**	**0.007**	**0.37 (0.10-1.56)**	**0.16**
**PDGFR-β Stromal Vessel**	**3803.74 (25.2)**	**0.12**	**1.86 (0.92-3.76)**	**0.08**
PDGFR-β Tumor Vessel	1060.14 (20.9)	0.005	0.93 (0.41-2.12)	0.86
PDGFR-β Stroma	6438.14 (15.1)	0.007	0.87 (0.33-2.60)	0.87
**Collagen I Tumor**	**853.82 (49.6)**	**0.001**	**1.61 (0.86-3.02)**	**0.13**
Collagen I Stromal Vessel	***2587.16 (25.2)***	***<0.001***	***2.31 (1.22-4.35)***	***0.008***
Collagen I Tumor Vessel	1653.24 (24.0)	<0.001	1.33 (0.65-2.72)	0.43
Collagen I Stroma	3285.71 (28.8)	0.001	1.36 (0.72-2.56)	0.34
**Collagen IV Tumor**	**467.87 (45.7)**	**<0.001**	**1.59 (0.84-3.01)**	**0.15**
Collagen IV Stroma Vessel	1832.17 (31.7)	0.005	1.28 (0.62-2.63)	0.51
Collagen IV Tumor Vessel	608.21 (48.9)	0.004	1.31 (0.69-2.48)	0.41
Collagen IV Stroma	2602.54 (14.0)	0.002	1.26 (0.49-3.22)	0.63
FN Tumor	5156.43 (52.3)	0.002	1.50 (0.77-2.92)	0.23

FN Stroma Vessel	5239.83 (55.8)	0.02	1.28 (0.65-2.53)	0.47
**FN Tumor Vessel**	**6119.94 (33.5)**	**0.004**	**1.82 (0.89-3.72)**	**0.10**
**FN Stroma**	**10380.35 (12.2)**	**<0.001**	**2.03 (0.72-5.75)**	**0.17**
HIF2α Tumor	1738.02 (31.8)	0.05	0.76 (0.35-1.66)	0.49
HIF2α Stromal Vessel	***1881.86 (26.1)***	***0.008***	***0.33 (0.13-0.86)***	***0.02***
**HIF2α Tumor Vessel**	**1054.97 (65.4)**	**0.20**	**0.61 (0.33-1.14)**	**0.12**
HIF2α Stroma	2005.59 (61.6)	0.08	0.86 (0.45-1.64)	0.65
CAIX Tumor	598.58 (88.4)	0.02	1.20 (0.37-3.90)	0.76
CAIX Stroma Vessel	2214.02 (13.4)	0.01	0.89 (0.23-3.12)	0.62
**CAIX Tumor Vessel**	**2641.25 (21.8)**	**0.002**	**0.52 (0.23-1.19)**	**0.16**
CAIX Stroma	2637.22 (15.7)	0.04	0.84 (0.37-1.91)	0.69

Table S2: Key antibody reagents. Provided is a list of key antibody reagents employed in this study with vendor, species, clone and final concentration provided.

Type	Name	Vendor	Species	Clone	Final Conc.
Target	TEM-1	Morphotek	Rat	9G5	1.04 µg/ml
	PDGFRβ	Cell Signaling	Rabbit	28E1	0.4 µg/ml
	Fibronectin-1	Abcam	Rabbit	Polyclonal	0.108 µg/ml
	Collagen I	Fitzgerald	Rabbit	Polyclonal	0.33 µg/ml
	Collagen IV	Fitzgerald	Rabbit	Polyclonal	0.33 µg/ml
	HIF2α	Novus Biologicals	Mouse	Ep190b	1.0 µg/ml
	CAIX	Morphotek	Rat	165F3	4.6 µg/ml
Compartment	Alexa488-Cytokeratin	eBioscience	Mouse	AE1/AE3	5 µg/ml
	Vimentin	Millipore	Chicken	Polyclonal	1:200 Serum
	CD31	DAKO	Mouse	JC70A	6.83 µg/ml
	CD31	Abcam	Rabbit	EPR3094	0.66 µg/ml
Secondary	Immpress Anti-Rat	Vector Labs	Horse	Polyclonal	Neat*
	Immpress Anti-Rabbit	Vector Labs	Horse	Polyclonal	Neat*
	Immpress Anti-Mouse	Vector Labs	Horse	Polyclonal	Neat*
	Mouse Envision Plus	DAKO	Goat	Polyclonal	Neat*

	Rabbit Envision Plus	DAKO	Goat	Polyclonal	Neat*
	Alexa Fluor 555 anti-chicken	Invitrogen	Goat	Polyclonal	1:200
	Alexa Fluor 750 Streptavidin	Invitrogen	Goat	Polyclonal	1:200

* As supplied by manufacturer

Table S3: TEM-1 assay precision analysis. Summary of day-to-day TEM-1 assay reproducibility with indicated Pearson's R correlations, slope, and % coefficient of variation (%CV).

Comparison	Pearson's R	Slope	%CV
Day 1 v. Day 2	0.973	0.999	4.12
Day 1 v. Day 3	0.950	0.997	
Day 2 v. Day 3	0.959	0.998	
